# Immunosuppressants/Immunomodulators and Malignancy

**DOI:** 10.3390/jcm14145160

**Published:** 2025-07-21

**Authors:** Norishige Iizuka, Yoshihiko Hoshida, Atsuko Tsujii Miyamoto, Hotaka Shigyo, Akira Nishigaichi, Gensuke Okamura, Shiro Ohshima

**Affiliations:** 1 Department of Pathology, Kishiwada City Hospital, Kishiwada 596-8501, Osaka, Japan; iizuka.n@gmail.com; 2 Department of Pathology, NHO Osaka Minami Medical Center, Kawachinagano 586-8521, Osaka, Japan; 3 Department of Respiratory Medicine and Clinical Immunology, Graduate School of Medicine, The University of Osaka, Suita 565-0871, Osaka, Japan; a.tsujii@imed3.med.osaka-u.ac.jp; 4 Department of Rheumatology and Allergology, NHO Osaka Minami Medical Center, Kawachinagano 586-8521, Osaka, Japan; hokaka306@gmail.com (H.S.); nishigaichi.akira.za@mail.hosp.go.jp (A.N.); ohshima.shiro.tr@mail.hosp.go.jp (S.O.); 5 Department of Orthopedics, NHO Osaka Minami Medical Center, Kawachinagano 586-8521, Osaka, Japan; gensuke.okamura@gmail.com

**Keywords:** post-transplant malignancy, post-transplant lymphoproliferative disorders, rheumatoid arthritis-associated lymphoproliferative disorders, rheumatoid arthritis-associated malignancy, methotrexate, immunosuppressive agents, anti-rheumatic agents

## Abstract

Individuals with immunosuppressive conditions are at a higher risk of developing malignancies than those in the general population. Immunosuppression weakens tumor immunity, hinders the detection of pro-oncogenic cells, and activates oncogenic viruses. Malignancies arising in immunosuppressed patients tend to be aggressive, have a high incidence of virus-associated cancers, and are reversible in some cases. Notably, immunosuppressive agents influence the frequency and type of malignancies, as well as their clinicopathological features. Organ transplant recipients receive long-term immunosuppressants to prevent acute rejection. Post-transplant malignancies vary depending on the type of drug administered before the onset of these diseases. Patients with rheumatoid arthritis (RA) are treated with long-term immunosuppressive medications, such as methotrexate (MTX). MTX is widely recognized as being associated with a specific type of lymphoproliferative disorder (LPD), known as MTX-associated LPD. Our recent report indicated that the clinicopathological features of rheumatoid arthritis-associated lymphoproliferative disorder (RA-LPD) also vary based on the other anti-RA agents used, such as tacrolimus and tumor necrosis factor inhibitors. Therefore, the clinicopathological characteristics of post-transplant LPD and RA-LPD evolve alongside the changes in the immunosuppressants/immunomodulators administered. Understanding the various characteristics and time trends of immunosuppressive neoplasms, particularly LPDs, in relation to different immunosuppressant/immunomodulator medications is highly valuable in clinical practice.

## 1. Introduction

Malignant neoplasms in patients with immunosuppressive conditions exhibit distinct characteristics compared to those in immunocompetent individuals [1,2,3,4]. This difference arises from the restoration of tumor immunity in the host following the reestablishment of defenses against tumors and oncogenic viral antigens [5]. A noteworthy aspect is the reversible nature of immunosuppressive neoplasms; spontaneous regression can occur after the administration, reduction, and alteration of the immunosuppressive agent [6]. This phenomenon has been observed in patients with various cancer types, including skin cancer, malignant melanoma (MM), Kaposi’s sarcoma (KS), lymphoproliferative disorder (LPD), hepatocellular carcinoma, and Merkel cell carcinoma [7,8,9,10,11].

The frequency and clinicopathological features of immunosuppressive neoplasms vary according to the specific immunosuppressive state [1,2,3,4]. Immunosuppressive conditions are categorized into inborn errors of immunity (congenital) and acquired forms; the latter is further divided into groups, including patients with acquired immunodeficiency syndrome (AIDS) and iatrogenic cases. The iatrogenic group includes solid organ transplant recipients and individuals with autoimmune diseases such as rheumatoid arthritis (RA), who are treated long-term with immunosuppressive agents [4,12]. Immunosuppressive agents have also enhanced the efficacy of organ transplantation over the years [13]. The introduction of powerful immunosuppressive agents may be associated with reduced survival rates as a result of the increased frequency and progression of malignant neoplasms [14,15,16]. Notably, the choice and dosage of immunosuppressive agent may influence the incidence and type of malignancy observed [1,2,4,17,18,19].

For patients with RA, the development of neoplasia, especially LPD, is associated not only with the immunosuppressive conditions induced by the anti-RA agents but also with the immune dysregulation inherent in RA itself [20]. For over half a century, patients with RA have been recognized as vulnerable to LPDs because of their hyperimmune state [21,22]. Epidemiological studies have indicated that the incidence of LPDs is two to four times higher in patients with RA before the introduction of methotrexate (MTX) as an immunosuppressive agent [23,24]. Baecklund et al. observed a correlation between LPD incidence and RA disease activity, emphasizing that diffuse large B-cell lymphoma (DLBCL) is more likely to develop in patients with RA [25,26]. Consequently, RA-LPDs, which arise from the immune dysregulation associated with RA, may be classified as disease-associated LPDs. Meanwhile, LPDs in RA patients receiving MTX have garnered significant attention since Elliman’s report in 1991 [27]. The 2001 World Health Organization (WHO) classification highlighted the immunosuppressive effects of MTX and was the first to define the terminology for MTX-associated LPD (MTX-LPD) [28]. In this context, RA-associated LPD (RA-LPD), linked to the immunosuppressive state caused by RA therapy, especially MTX, should be recognized as a therapy-related LPD. Thus, RA-LPD encompasses both disease- and therapy-related LPDs.

Over the past 30 years, significant advancements in the understanding of RA pathogenesis have ushered in a new era of treatment [29,30,31,32]. Previously, RA management primarily involved the use of prednisolone (PSL) and non-steroidal anti-inflammatory drugs (NSAIDs). The emergence of conventional synthetic disease-modifying anti-rheumatic drugs (csDMARDs) in the 1990s, such as salazosulfapyridine (SASP), led to the introduction of MTX and tacrolimus (TAC) as immunosuppressive agents [33]. MTX at a low dose causes immunosuppression by disrupting pyrimidine and purine synthesis and increasing adenosine levels, while TAC causes immunosuppression by blocking calcineurin. This was followed by a series of biologic DMARDs (bDMARDs), including anti-tumor necrosis factor (TNF)-α biologics such as infliximab (IFX), etanercept (ETN), adalimumab (ADA), golimumab (GLM), and certolizumab (CZP), and non-anti-TNF-α biologics, including tocilizumab (TCZ), which targets the interleukin (IL)-6 receptor, and abatacept (ABT), which uses CTLA4-Ig. Subsequently, targeted synthetic DMARDs (tsDMARDs) based on Janus kinase (JAK) inhibitors (JAKi), such as tofacitinib (TOF), upadacitinib (UPA), and baricitinib (BAR) were developed [29,30,31,32].

Recently, our group reported that the clinicopathological characteristics of RA-LPD vary based on the type and frequency of the anti-RA agent used, and that these characteristics change over time, resembling the effects of post-transplant malignancies [34]. This article mainly describes the malignancies that develop under immunosuppressive and immune dysregulated conditions, highlighting how the immunosuppressants/immunomodulators affect the post-transplantation-LPD (PT-LPD) and RA-LPD.

## 2. Immunosuppressive Agents and Organ Transplantation

### 2.1. Post-Solid Organ Transplantation Malignancy and Immunosuppressive Agents (Figure 1)

Patients who receive solid organ transplants develop malignant tumors at a rate that is two to four times higher than in the general population [1,5,17]. Malignant neoplasms in transplant recipients tend to display a more aggressive clinical course, with a rising prevalence of infection-related cancers such as human papillomavirus (HPV)-associated skin cancer and Epstein–Barr virus (EBV)-related lymphoma than in the general population.

According to a cohort report of 175,732 transplants in the United States from 1987 to 2008, the standard incidence ratios (SIRs) for developing malignancies in transplant recipients were significantly elevated for most infection-related malignancies compared to the general population, including non-Hodgkin lymphoma (NHL) (SIR: 7.54), Kaposi sarcoma (SIR: 61.46), and cancers of the liver (SIR: 11.56), anus (SIR: 5.84), and vulva (SIR: 7.60). Among infection-unrelated malignancies, SIRs were significantly elevated for lung (SIR: 1.97), kidney (SIR: 4.65), skin (non-melanoma, non-epithelial) (SIR: 13.85), lip (SIR: 16.78), and intrahepatic bile duct cancers (SIR: 5.76). In contrast, the risk of breast cancer (SIR: 0.85) and, to a lesser extent, prostate cancer (SIR: 0.92) decreased [1].

The type of cancer varies based on the transplanted organ and the immunosuppressive agents used [1,2,4,17,18,19]. 

Immunosuppressive therapy is vital for preventing organ transplantation rejection. Historically, immunosuppressive agents have been improved to decrease rejection rates and enhance the retention of the transplanted organs. In the 1960s, the use of maintenance immunosuppressive agents in organ transplantation began with azathioprine (AZ). In the 1980s, calcineurin inhibitors (CNI) such as cyclosporine A (CyA) and TAC were introduced, followed by mycophenolate mofetil (MPA) and mycophenolate (MMF). In the 2000s, everolimus (EVL) and sirolimus (SRL) emerged as a mammalian target of rapamycin (mTOR) inhibitors [35]. Considering these factors, the history of post-transplant malignancies can be classified into three eras based on the type of immunosuppressant used for the maintenance therapy [36]; these are outlined below.

#### 2.1.1. Azathioprine (AZ) Era

Since AZ was approved by the Food and Drug Administration (FDA) in 1963, the AZ era began in the 1960s and lasted until the 1970s. AZ is widely used as a maintenance immunosuppressant in transplantation medicine. It suppresses the immune system by inhibiting purine, DNA and RNA synthesis, and T-cell proliferation [36]. Additionally, AZ has been reported to be carcinogenic in cases where the DNA in the epidermal cells is mutated due to ultraviolet exposure, as it directly inhibits the DNA repair mechanisms [37,38].

An epidemiological study of the AZ era reported on 693 cases of malignant tumors in the Denver Transplant Tumor Registry (DTTR) prior to August 1978, before the introduction of CyA. The most common types of malignant tumor were skin and lip cancer (40.0%), followed by LPD (19.2%), cervical cancer (7.1%), lung cancer (5.2%), colorectal cancer (3.0%), and KS (2.9%) [39]. A 2016 meta-analysis also showed that transplant patients who received AZ had a higher incidence of squamous cell carcinoma of the skin than those who did not (odds ratio (OR) = 1.56; 95% confidence interval (CI): 1.11–2.18) [40].

#### 2.1.2. Calcineurin Inhibitor (CNI) Era

The 1980s and the 1990s were the era of CNIs. In 1983, CyA was approved by the FDA for immunosuppression in transplantation, and TAC was approved in 1994. These medications are now widely used in transplantation. CyA binds to cyclophilin A, whereas TAC binds to the intracellular protein FKBP12. In both of these CNIs, their binding forms a complex consisting of Ca^2+^, calmodulin, and calcineurin, which inhibits the phosphatase activity of calcineurin. This inhibition prevents the dephosphorylation and nuclear translocation of NFATs, thereby inhibiting the production of IL-2. As a result, CNIs inhibit T- cell activation and proliferation, contributing to immunosuppression [35,36].

CNIs also act as carcinogens by increasing transforming growth factor (TGF)-β1 [41,42], a potent inhibitor of IL-2-stimulated T-cell proliferation, and by stimulating the tumor angiogenesis factor vascular endothelial growth factor (VEGF) [41]. Additionally, the elevated IL-6 levels in EBV-infected B-cells promote the activation, proliferation, and possibly the immortalization of these cells [43,44].

The frequency of malignant tumors was higher in the CyA group than in the AZ group [18]. Penn reported that, among 773 CyA monotherapy regimen transplants in the CTTR until March 1995, the most common types of malignant tumor were skin and lip cancer (25.1%), followed by lymphoma (22.5%), KS (8.4%), kidney cancer (6.5%), cervical cancer (1.6%), and vulvar/perineum cancer (0.6%) [45]. Compared with the AZ therapy, the incidence of lymphoma, KS, and renal cancer increased, whereas the incidence of squamous cell carcinoma (SCC), cervical cancer, and vulvar cancer decreased [46]. A report from Japan published in the same era also showed that the CyA group had a lower frequency of SCC, no KS, and a higher frequency of kidney and thyroid cancers than the non-CyA group, which consisted mainly of AZ-treated patients [18]. Compared with TAC and CyA, OPTN/UNOS data indicated that TAC significantly reduced the incidence of all non-skin cancers, non-lymphoma solid tumors, and non-MM skin cancers compared with CyA [36,47].

#### 2.1.3. Mammalian Target of Rapamycin (mTOR) Inhibitor Era

Since the start of the new millennium, new immunosuppressants have become available for use in transplantation. SRL was approved by the FDA in 1999, marking the beginning of the era of mTOR inhibitors in 2000, followed by the approval of EVL in 2010. Both are representative mTOR agents [48]. The immunosuppressive activity of the mTOR inhibitors is based on their binding to immunophilin (FKBP12), which downregulates p70S6 kinase activity and blocks the IL-2 stimulation of lymphocyte proliferation [49].

**Figure 1:**
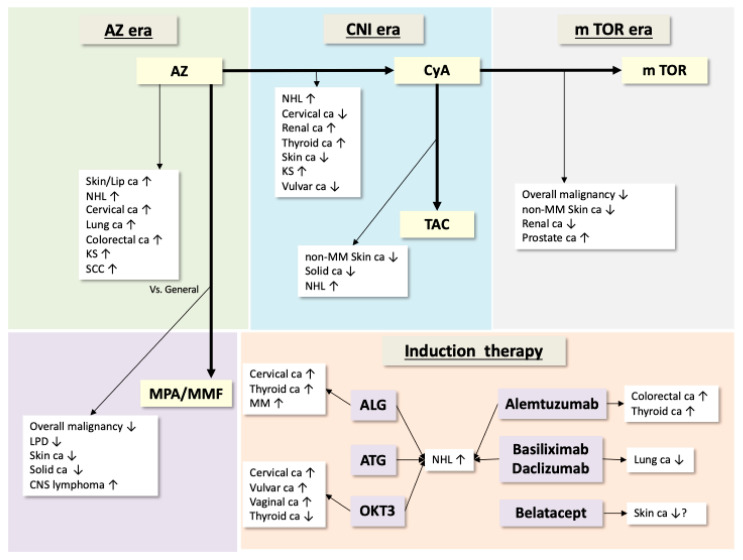
Changing patterns of post-transplant malignancy according to immunosuppressive agent [2,14,15,18,36,39,40,45,46,47,50,51,52,53,54,55,56,57,58,59,60,61,62]. The type of cancer depends on the immunosuppressive agents used before the development of the malignancies. As immunosuppressive therapies have advanced, the patterns of malignant tumors in transplant patients have changed, as shown in this figure. Abbreviations: AZ: azathioprine, ca: carcinoma, NHL: non-Hodgkin lymphoma, KS: Kaposi’s sarcoma, SCC: squamous cell carcinoma, MPA: mycophenolate mofetil, MMF: mycophenolate, LPD: lymphoproliferative disorders, CNS: central nervous system, CNI: calcineurin inhibitors, CyA: cyclosporine A, TAC: tacrolimus, MM: malignant melanoma, mTOR: mammalian target of rapamycin, ALG: anti-lymphocyte globulin, ATG: anti-thymocyte globulin.

mTOR demonstrates anti-tumor effects by inhibiting cell proliferation through the PI3 kinase [63] and p70S6 kinase pathways as a result of the deletion of PTEN. It induces growth arrest by upregulating E-cadherin, enhancing the expression of cell cycle inhibitors (e.g., p27kip1) [64], decreasing IL-10 secretion, preventing the constitutive activation of signal transducer and activator of transcription (STAT)1 and STAT3 [65], and disrupting VEGF production, which hinders the VEGF-induced stimulation of vascular endothelial cells [49,66].

Although the risk of post-transplant MM has increased significantly over the last decade, a meta-analysis revealed that the overall incidence of all malignancies and non-MM skin cancers has decreased [2,50,51,52,53]. Another study reported a decline in renal cancer, alongside an increased incidence of prostate cancer, when compared with CyA [2,53,54].

#### 2.1.4. Mycophenolic Acid (MPA)/Mycophenolate Mofetil (MMF)

Mycophenolic acid (MPA) and mycophenolate mofetil (MMF) were approved for kidney transplantation in 1995 and are currently widely used as alternatives to AZ. Both agents are combined with CNIs or mTOR inhibitors for maintenance therapy. They exert immunosuppressive effects as purine synthesis inhibitors that target inosine monophosphate dehydrogenase (IMD) [14,67], reducing the incidence of malignant tumors by inhibiting IMD proliferation, which is increased in tumor cells. However, sufficient immunosuppression is also thought to decrease the need for potent immunosuppressive agents [68].

Compared with other immunosuppressive treatments, MMF significantly reduces the incidence of PT-LPD, total cancer, skin cancer, and solid tumors [14,36,55]. However, as discussed later, MMF administration increases the incidence of CNS lymphoma [56,57].

#### 2.1.5. Immunosuppressants for Transplant Induction Therapy

Transplant induction therapy is a short-term medication used during transplant induction or in cases of acute rejection. Immunosuppressive agents for this therapy are classified as polyclonal antibodies (anti-lymphocyte globulin (ALG)) and monoclonal antibodies. The former includes thymoglobulin (RATG, FDA approved in 1999) and anti-thymocyte globulin (ATG, approved in 1998), while the latter encompasses OKT3 (anti-CD3 antibodies, approved in 1985), alemtuzumab (anti-CD52, approved in 2014), basiliximab (anti-CD25, IL-2α receptor, approved in 1998), daclimuzab (anti-CD25, IL-2α receptor, approved in 1997, discontinued in 2009), and belatacept (anti-CD80/86, approved in 2011) [58,69,70,71,72,73,74,75,76].

All antibody induction therapies, including ALG, ATG, OKT3, alemtuzumab, basiliximab, and daclizumab, increase the frequency of PT-LPD [15,58,59,60]. In addition to lymphoma, ALG therapy elevates the incidence of colorectal cancer, thyroid cancer, and MM [58,59]. OKT3 therapy increases the incidence of cervical, vaginal, and vulvar cancers [59,61] and decreases the incidence of thyroid cancer [58]. The anti-IL-2α receptor treatments, namely, basiliximab and daclizumab, reduce lung cancer incidence. Alemtuzumab is linked to a higher incidence of colorectal and thyroid cancers [58]. Belatacept has been reported to lower the risk of skin cancer [62]; however, recent studies have reported contradictory results [77].

### 2.2. Post-Transplantation Lymphoproliferative Disorders (PT-LPD) and Immunosuppressive Agents (Figure 1)

Post-transplantation lymphoproliferative disorders (PT-LPDs) are the second most common malignant neoplasms that arise following organ or bone marrow transplantation, comprising clinically and morphologically diverse lymphoid proliferative disorders [4]. PT-LPDs are histologically classified into four groups: non-destructive PT-LPDs, which includes reactive plasmacytic hyperplasia; polymorphic PT-LPDs, monomorphic PT-LPDs, and other types, including plasmacytoma-like PT-LPDs. PT-LPDs are mainly associated with EBV and tend to have an aggressive clinical course. While PT-LPDs mainly involve B-cells, T-cells, and natural killer (NK) cells, Hodgkin lymphoma (HL) can also occur [78,79,80]. A noteworthy aspect is the reversible nature of PT-LPDs, as first reported by Starzl et al. [6]. Non-destructive LPDs and the majority of polymorphic PT-LPDs regress spontaneously after the discontinuation of immunosuppressive agents [81].

In this section, we discuss the types of immunosuppressants and the clinicopathological characteristics of PT-LPDs.

### 2.3. Central Nervous System (CNS) Lymphoma

Central nervous system (CNS) involvement frequently occurs in immunosuppressive LPD cases. Various immunodeficiency conditions and immunosuppressive drugs affect the frequency of CNS-LPD. Before the advent of combination antiretroviral therapy (cART), primary CNS lymphomas were more prevalent among HIV-positive patients. Since the introduction of cART, the frequency of non-Hodgkin lymphoma (NHL) has decreased by 50% [82]. CNS-LPD is a common post-transplant malignancy among patients receiving Aza, MMF, and belatacept regimens [56,57,83,84,85]. However, it has become rare since the introduction of CyA [18]. Conversely, it has also been suggested that CyA treatment may suppress CNS-LPD.

### 2.4. Time Trends According to Changes in Immunosuppressants

Since 1983, CyA has been widely used for transplantation instead of AZ. In addition to the decreased frequency of CNS lymphoma, changes in the characteristics of PT-LPD have been observed. PT-LPDs in patients receiving the CyA regimen occur earlier after transplantation, with increased rates of widespread lymph node involvement, gastrointestinal tract issues, and a higher regression rate following the reduction or cessation of immunosuppressive therapy when compared to those receiving AZ [46]. There were better prognoses in the CyA era than in the AZ era [80].

In 1994, TAC was introduced into transplant medicine and became widely used in the field. Regarding LPD, although reports suggest that its administration increases the incidence of LPD [15,43,86,87], no differences were found between the two groups in terms of LPD incidence or clinicopathological features compared to the TAC and CyA groups [14]. At the outset of TAC’s introduction, concerns emerged that it could elevate the incidence of PT-LPD, particularly in pediatric liver transplantation [88,89,90,91]. According to a report from Pittsburgh, the incidence of PT-LPD was the same for CyA and TAC; however, the incidence of PT-LPD in pediatric liver transplantation tended to be higher for TAC than for CyA (*p* = 0.06) [86]. Nevertheless, in 2013 Narkewicz et al. examined the incidence of PT-LPD in pediatric liver transplantation over a 12-year period (1995–2007) and reported a decrease in PT-LPD incidence. This decrease was attributed to weight loss resulting from immunosuppressant dosing and the monitoring of EBV DNA levels in the blood [89].

A 2011 report from Sweden indicated a statistically significant decrease in the NHL risk among non-kidney transplant recipients from 2000 to 2008 compared to those from 1990 to 1999 [88]. Tsai et al. observed a shift in the histological type and the time to onset of PT-LPD, as documented over time using the UNOS OPTN database from 1999 to 2013. They identified that while the proportion of polymorphic LPD (P-LPD) decreased, the proportion of monomorphic LPD (M-LPD) increased, with an extended time to onset. This change was attributed to the increased use of TAC and a possible increase in the transplant patients’ survival rates [92].

The EBV positivity rate also appears to have changed over time. Nelson noted that the proportion of EBV-negative PT-LPD was 2% until 1991 but rose to 23% during the 1990s [93]. EBV-negative PT-LPD has distinct genetic features compared to EBV-positive PT-LPD and is genetically similar to de novo lymphoma [94]. Luskin also indicated that the percentage of EBV-negative PT-LPD increased from 10% in 1990–1995 to 48% in 2008–2013 [95]. Leblond reported that the median time between organ transplantation and tumor diagnosis for EBV-negative PT-LPD was 1800 days, compared to 180 days for EBV-positive LPD; thus, EBV-negative PT-LPD may be a late complication of organ transplantation, induced by the different regimens of immunosuppressive agents [96].

## 3. Anti-RA Agents and Malignancy

### 3.1. Malignancy in Patients with RA and Anti-RA Agents

RA is an autoimmune disorder marked by persistent inflammation in the joints and synovium, leading to joint damage and the loss of function, along with the production of autoantibodies, including rheumatoid factor (RF) and anti-citrullinated protein antibody (ACPA) [97,98]. The incidence of malignant tumors in patients with RA has been reported to be either higher or lower than that in the general population, making this point epidemiologically controversial [33,99,100,101]. However, the frequency of immune dysregulation and the use of anti-RA agents in patients with RA differ from those in the general population for each type of malignancy.

Patients with RA have a higher risk of developing lymphoma and lung cancer but a lower risk of developing colorectal and breast cancers than the general population [33,102,103,104]. Additionally, a recent report from France highlighted a significant increase in the incidence of bladder cancer, cervical cancer, prostate cancer, MM, DLBCL, multiple myeloma, and HL. In contrast, the incidence of pancreatic, breast, and endometrial cancers is low [105]. Other studies have indicated a high incidence of leukemia, particularly acute myeloid leukemia (AML) [106], and non-MM skin cancer [107,108]. Conversely, other studies have reported a low incidence of liver [33] and prostate cancers [109].

The pathogenesis of neoplasia in patients with RA may arise from the immune dysregulated state inherent to RA or from an immunosuppressive state induced by antirheumatic agents [20]. It is well established that the hyperimmune state in RA contributes to the increased frequency of LPD [21,22], and that these LPDs can be classified as disease-associated LPDs. Furthermore, the increased frequency of lung cancer has been linked to a hyperimmune state associated with interstitial lung disease and smoking. Moreover, the influence of anti-rheumatic agents on malignant tumor development should not be overlooked.

Anti-RA agents have advanced significantly over the past 30 years, providing substantial benefits to RA patients [29,30,31]. Traditionally, anti-inflammatory PSLs and analgesic NSAIDs have been used to alleviate the pain associated with RA. Additionally, SASP was approved by the FDA in 1950 as an immunomodulator, followed by the introduction of cDMARDs, with MTX, leflunomide (LEF), and TAC being approved as immunosuppressants in 1988, 1998, and 2005, respectively. Subsequently, bcDMARDs for RA were introduced, including the soluble TNFi ETN (approved in 1998); TNFi monoclonal antibodies such as IFX (approved in 1999); ADA (approved in 2002); GLM (approved in 2009); CZP (approved in 2009); rituximab (RTX) (approved in 1998), which targets the anti-CD20 antibody; ABT (approved in 2005), which utilizes CTLA4-Ig; and TCZ (approved in 2010), which targets the IL-6 receptor. Furthermore, TOF (approved in 2012), upadacitinib (UPA) (approved in 2019), and baricitinib (BAR) (approved in 2022) which are small-molecule compounds classified as JAKis, have also been employed as tsDMARDs [29,30,31,32,110].

#### 3.1.1. Non-Steroidal Anti-Inflammatory Drugs (NSAIDs)

Traditionally, anti-inflammatory PSLs and analgesic NSAIDs have been used to alleviate the pain associated with RA [29]. The selective inhibition of cyclooxygenase-2 by NSAIDs suppresses the production of prostaglandins, thereby preventing the incidence of colorectal, breast, lung, and prostate cancers [111,112,113]. Recent reports from Japan indicate that the incidence of colorectal cancer significantly decreased in the 2000s; however, this notable difference disappeared in the early 2010s [114]. These phenomena are believed to result from the reduced frequency of NSAID use among patients with RA due to the advancements in RA treatment drugs, as suggested [33]. The lower risk of developing colorectal and breast cancers is thought to be due to the use of NSAIDs [99,112,115].

#### 3.1.2. Conventional Synthetic DMARD (csDMARD)

##### Methotrexate (MTX)

MTX is now recognized as an anchor drug for RA [29,30,31,32,33]. Methotrexate is a folate analog that inhibits dihydrofolate reductase, thereby hindering DNA synthesis and inducing cell death. The anti-inflammatory effects of MTX may involve mechanisms beyond dihydrofolate reductase inhibition. Specifically, these include suppressing cell proliferation by inhibiting purine metabolism, increasing the rate of T-cell apoptosis by inhibiting T-cell activation, enhancing endogenous adenosine release, inhibiting the production of cytokines and intercellular adhesion molecules, and blocking IL-1β receptor binding [116].

The tumorigenic effect of MTX may arise from an immunosuppressive state that reduces tumor immunity. Feng et al. [117] suggested that the unique impact of MTX, which induces EBV replication while promoting immunosuppression, could explain its association with the development of EBV-LPD in RA patients. Regarding the direct carcinogenicity of MTX, it has been reported to exhibit chromosomal cytogenetic effects [20]; however, no further findings have been reported.

The statistical evidence surrounding the overall risk of malignancies, including LPD, in patients with RA treated with MTX remains controversial. Although the incidence of non-MM skin cancer is reportedly increased, many studies have not reported that LPD risk is associated with MTX treatment [26,118,119]. Conversely, some studies have indicated a significant increase in LPD incidence with MTX treatment [33,120,121]. Owing to its characteristic clinicopathological findings, such as the regression of the LPD after MTX cessation, it was designated MTX-LPD in the 2001 WHO classification [28]. These findings support an association between MTX and the development of LPD, which is categorized as a treatment-related LPD. The LPD is discussed in the next section.

##### Tacrolimus (TAC)

The immunosuppressive effects and carcinogenic properties of TAC were discussed in the section on PT-LPD [36]. Several studies have reported a significant association between TAC administration and the occurrence of malignancies in patients with rheumatoid arthritis [33,122].

##### Other csDMARD

SASP and LEF do not appear to be associated with cancer risk [116].

#### 3.1.3. Biologic DMARDs (bDMARDs)

##### Tumor Necrosis Factor Inhibitors (TNFis)

TNF-α was initially identified as a factor that induces tumor necrosis and was subsequently shown to act as a carcinogenic promoter in chronic inflammation. TNF-α activates downstream signaling pathways, including NF-κB, leading to inflammation and the induction of cancer-associated chemokines, cytokines, growth factors, and angiogenesis. Additionally, TNF-α binds to TNF receptor 1, forming the TNFR-associated death domain/Fas-associated death domain complex, which activates caspase-8 and induces apoptosis in cells. Tumor necrosis factor inhibitors (TNFis) not only exhibit anti-inflammatory and anti-tumor effects but also promote tumor growth by inhibiting apoptosis [123].

Most previous studies have reported no significant increase in the incidence of malignancies overall following TNFi treatment [119,124,125,126,127,128,129,130,131,132,133,134,135,136]. However, meta-analyses conducted soon after the introduction of TNFis indicated an increased incidence of these events [137]. In contrast, other studies have indicated a decrease in incidence [133,138]. Several studies have reported an increase in LPDs [119,137,139], as well as in instances of non-MM skin cancer [128,135,140], MM [141], and urinary tract cancer [138], but not in other solid tumors. The relationship between LPD and TNFi treatment is discussed in the following section.

##### Rituximab (RTX)

RTX is a CD20 antibody that depletes circulating mature B lymphocytes [142]. This reduction in antigen presentation by the B-cells leads to T-cell activation. A prolonged period of rituximab-induced B-cell depletion may compromise the immune system, including tumor immuno-surveillance [143]. Since 2000, RTX has been used in combination with CHOP therapy for the treatment of DLBCL. However, a report from California, USA, investigated the occurrence of secondary cancers before and after combination therapy with RTX and DLBCL and revealed that the incidence of AML and thyroid cancer increased after RTX introduction [144]. Furthermore, several studies have shown that the overall incidence of malignant tumors was increased [145].

Patients with RA treated with RTX face an increased overall risk of malignancy, particularly lung, bladder, urinary tract, otopharyngeal, and head and neck cancers, as well as HL and MM, while demonstrating a decreased risk of breast and endometrial cancers compared with the general population [101,136]. However, Huss reported that RTX was not related to the overall incidence of malignancy [136].

##### Abatacept (ABT)

ABT is a fusion protein composed of the extracellular domain of human CTLA-4, which selectively modulates the co-stimulation of T lymphocytes by inhibiting their activation through binding to CD80/CD86, thereby preventing interaction with the CD28 expressed on T-cells [29].

ABT therapy is associated with an overall increased risk of malignancies in patients with RA [101,136,146]. Compared to the general population, the incidence of lung, bladder, and urinary tract cancers is higher [101,136]. However, other studies have not found a significant difference in the comparison of patients with RA treated with other DMARDs [147,148,149,150,151]. Nonetheless, ABT treatment was associated with a slightly elevated risk of non-melanotic skin cancer and malignant melanoma compared to other DMARDs [146,147,148,149,152]. The increased incidence of melanoma is consistent with the widespread use of the CTLA-4 inhibitor ipilimumab for MM treatment [153].

##### Tocilizumab (TCZ)

Tocilizumab (TCZ) blocks IL-6-mediated signaling by preventing IL-6 from binding to both the transmembrane and soluble forms of the IL-6 receptor. IL-6 plays a crucial role in regulating immunity and inflammation [154] by activating the JAK2 and STAT3 signaling pathways. The activation of the JAK2/STAT3 signaling pathway contributes to tumorigenesis. It promotes the formation of an inflammatory microenvironment in tumors and is closely associated with the development and progression of various cancers. Therefore, IL-6 inhibitors are being studied for their potential application as key molecules in cancer therapy that target the IL-6/JAK2/STAT3 signaling pathway [155].

Compared to the general population, TCZ therapy does not appear to be associated with an increased overall risk of malignancies in patients with RA [101,137,156]. However, the incidence of lung, bladder, and cervical cancers has increased, whereas that of breast cancer has decreased [101,157].

#### 3.1.4. Targeted Synthetic DMARDs (tsDAMRDs)

##### Janus Kinase Inhibitors (JAKis)

JAKs are tyrosine kinases that play a role in cytokine receptor signaling. JAK regulates the transcription of several genes associated with inflammatory, immune, and cancerous conditions [32]. The excessive activation of JAK and its associated JAK/STAT signaling promotes cancer hallmarks such as proliferation, survival, and angiogenesis, in both the tumor and its surrounding environment, through the activation of STAT3, IL-6, IL-11, IL-22, IL-23, and interferon-γ (IFN-γ) [158]. Inhibiting JAK may provide an alternative cancer treatment [159]; a JAKi has been approved as an anticancer therapy for myelofibrosis [160]. However, JAKs also prevent tumor progression by activating CD8-positive T-cells and natural killer cells, raising the crucial question of whether JAKis increase the risk of malignancy.

TOF primarily targets ATP-binding sites, specifically JAK1 and JAK3, disrupting cytokines such as IL-2, IL-6, type 1 IFNs, and IFN-γ [161]. BAR selectively binds to the ATP-binding sites of JAK1 and JAK2, whereas UPA selectively binds to JAK1 [32]. Although many reports suggest that there is no significant increase in the incidence of malignancies with TOF, UPA, and BAR [162,163,164,165,166,167,168], others have documented a noticeable increase in the frequency of malignancies compared to that in patients treated with TNFis [169,170,171,172,173], thereby the FDA and European Medicine Agency (EMA) emitted warnings in 2021 and 2023, respectively [174]. This is particularly evident in patients aged ≥ 50 years, who are at risk of cardiovascular disease [169]. The incidence of non-MM skin cancer and lung cancer is reportedly higher in patients receiving JAKis than in those treated with TNFis [163,169,175].

### 3.2. Time Trends of Malignancy in Patients with RA

Comparisons between two meta-analyses conducted in Canada in 2008 and the United States in 2015 can be utilized to understand the time trends of malignancy in patients with RA [99,102]. The 2008 meta-analysis conducted in Canada did not confirm an increased overall risk of malignant tumors in patients treated with DMARDs. In contrast, the 2015 report indicated that the overall incidence of malignant tumors, particularly lymphomas, was higher in patients than in the general population. However, no differences were observed in the incidence of malignant melanoma, cervical cancer, or prostate cancer between the two groups.

According to the Japanese IORRA cohort [103], the period from 2000 to 2013 was divided into three phases: the pre-biological agent period (the early 2000s, 2000–2004), the biological agent period (the late 2000s, 2005–2009), and the late biological agent period (the early 2010s, 2010–2013). Changes in malignant tumors over time have been reported. The report indicated that the frequencies of all malignant tumors and lymphomas remained unchanged. However, while the incidence of breast cancer was not significantly different between patients with RA and the general population in the 2000s, it decreased significantly in the early 2010s. Furthermore, although the incidence of lung cancer was considerably higher in the early 2000s, this disparity decreased from the late 2000s to the early 2010s. These results suggest that while malignant tumors and lung cancer are experiencing a downward trend compared to the general population, the incidence of colorectal cancer is increasing. Although the incidence of malignant lymphoma is decreasing, it remains significantly higher than in the general population.

Factors contributing to the decrease in lung cancer patients with rheumatoid arthritis include a reduction in the smoking population, decreased chronic inflammation due to interstitial pneumonia, and the influence of a downward trend in CRP levels [176]. The decline in lymphoma, particularly DLBCL, may be attributed to the reduced disease activity associated with improved treatment. This advancement has resulted in a decrease in the incidence of disease-related LPD. Furthermore, a heightened awareness of MTX-LPD and the widespread use of MTX alternatives may contribute to the occurrence of treatment-related LPD.

## 4. Rheumatoid Arthritis Associated Lymphoproliferative Disorders (RA-LPDs) and Anti-RA Agents (Figure 2)

RA-LPD has distinct characteristics that differentiate it from general lymphomas. It occurs in approximately 1 in 2000 patients with RA, and its frequency is two to four times higher than that in the general population [23,24,114]. According to our database, the male-to-female ratio was 1:2.24, indicating a predominance in females, with a median age at disease onset of 70 years. The median duration from RA onset to LPD onset was 12 years, and the median time from the first administration of MTX to LPD onset was just over five years. The EBV-encoded small RNA-1 (EBER-1) positivity rate was notably high at 51.8%, and C-reactive protein (CRP) and lactate dehydrogenase (LDH) levels were elevated at the onset of LPD [34].

All histological types of malignant lymphomas are present in RA-LPD; however, immunodeficiency-associated LPD can also occur. The most common histological type is DLBCL, followed by HL, mucosa-associated lymphoid tissue lymphoma (MALToma), P-LPD, follicular lymphoma (FL), angioimmunoblastic lymphoma (AITL), HL-like lesions, and EBV-positive mucocutaneous ulcer (EBV-MCU). Compared to sporadic LPD, RA-LPD is characterized by the presence of immunodeficiency lymphomas, such as P-LPD, HL-like lesions, and EBV-MCU, along with an increased proportion of HL, P-LPD, and AITL. Conversely, MALToma, FL, and T-cell phenotypes, such as peripheral T-cell lymphoma (PTCL), anaplastic large cell lymphoma (ALCL), and adult T-cell leukemia/lymphoma (ATLL), are less prevalent [34].

In our database, RA-LPD regressed in 81.6% of cases within 2 weeks to 3 months after the discontinuation of MTX. In 32.1% of the regressions, re-growth occurred at a median of 1 year (1–92 months). The 5-year remission maintenance rate (5y-PregFS) after regression following MTX discontinuation was 63.8%. In contrast to PT-LPD, the proportion of cases achieving CR owing to treatment effects was high. The tumor death rate was 16.3%, and the 5-year survival rate was 86.3%, indicating that the prognosis is better than that for sporadic LPD [34].

### 4.1. Pre-MTX Era Conventional Therapy and RA-LPD

Before MTX use, patients with RA were treated with medications such as SASP, BCL, sodium aurothiomalate, and PSL. This group can be considered to have disease-related LPD affected by the disease activity of RA without the influence of anti-RA drugs, and an increased frequency of DLBCL has been reported [25,26]. In our database, this naïve group showed a lower frequency of EBER-1 positivity than the other groups. Extranodal origins, particularly from the breast, stomach, and orbit, as well as the B-cell phenotype, including MALToma and FL, may characterize the original nature of RA-LPD. Similar to PT-LPD [34], indolent lymphomas such as FL and MALToma may not be immunosuppressive LPDs in RA-LPD.

#### 4.1.1. Conventional Synthetic DMARD (csDMARD) and RA-LPD

MTX is now recognized as a cost-effective cornerstone of RA therapy [28,29,30,31]. The MTX group is prominent, and numerous recent studies have highlighted its clinicopathological features [4,28,34,120,177,178,179,180,181,182,183,184,185,186,187,188]. This is also detailed in the WHO classification [4,28,82]. In our database, a comparison of the MTX monotherapy group with the naïve group elucidated the pathology of pure MTX-LPD. The MTX monotherapy group had a higher proportion of primary oral lesions and a lower proportion of primary orbital or gastric lesions and MALTomas than the naïve group. In the MTX monotherapy group, the incidence of oral primary lesions increased, whereas that of orbital or gastric primary lesions and MALTomas decreased.

Regarding TAC, a comparison between the MTX plus TAC combination and MTX monotherapy groups revealed a significant increase in the proportion of primary lesions and ALCL in the pharyngeal and parotid glands, along with a decrease in the proportion of primary lesions in the lungs [34]. TAC also affects lymphoma formation in RA-LPD. We allowed overlap with our other drugs and compared 78 of the 518 patients with RA to 78 patients receiving TAC and 440 patients without TAC. Consequently, an increase in EBER-1 positivity, P-LPD subtype, and advanced age at LPD onset were identified as independent poor prognostic factors in patients treated with TAC therapy [123]. Patients receiving TAC medications for RA may require a detailed follow-up to assess LPD development.

#### 4.1.2. Biologic DMARDs (bDMARDs) and RA-LPD

A TNFi does not increase the incidence of LPD, as described above in the relationship between TNFi administration and the development of LPD [125,126,127,128,129,130,131,132,133,134,135]. Although the FDA expressed concern about LPD development in the early stages of TNFi administration [119,139,189], following several studies reporting cases of LPD, particularly within the first few months of TNFi treatment [190], no similar reports have yet been published.

**Figure 2:**
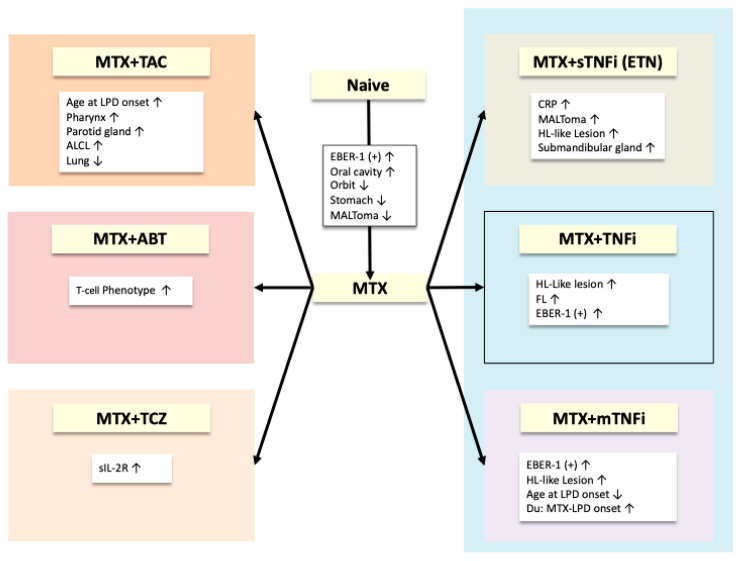
Changing patterns of rheumatoid arthritis-associated lymphoproliferative disorders according to anti-rheumatic agent [34,191] The clinicopathological features of rheumatoid arthritis-associated lymphoproliferative disorder (RA-LPD) also differ based on the anti-RA agent used, including methotrexate, tacrolimus, and tumor necrosis factor inhibitors, among others, as illustrated in this figure. Abbreviations: MTX: methotrexate, TAC: tacrolimus, LPD: lymphoproliferative disorder, ALCL: anaplastic large cell lymphoma, ABT: abatacept, TCZ: tocilizumab, sIL-2R: soluble interleukin-2 receptor, EBER-1: Epstein–Barr virus-encoded small RNA-1, MALToma: mucosa-associated lymphoid tissue lymphoma, ref.: reference, sTNFi: soluble tumor necrosis factor inhibitor, ETN: etanercept, CRP: C-reactive protein, HL-like lesion: Hodgkin lymphoma-like lesion, TNFi: tumor necrosis factor inhibitor, FL: follicular lymphoma, mTNFi: monoclonal tumor necrosis factor inhibitor, MTX-LPD: methotrexate-associated lymphoid tissue lymphoma, Du: duration.

The incidence and histologic subtypes of the LPDs depend on each anti-RA agent, including TNFis, and have recently garnered more interest [192]. Although previous studies have not shown any correlation with the histological subtypes of LPDs [130,186,193], our database allowed us to statistically demonstrate the effect of TNFi administration on LPD. The MTX plus TNFi combination groups were compared with the MTX monotherapy group. In the MTX plus TNFi groups, there was an increase in EBER-1-positive cases, a higher proportion of HL-like lesions, and a decrease in the proportion of FL than in the MTX monotherapy group [34].

TNF is a trimeric protein produced by various cells and exists in both soluble and membrane-bound forms [194]. Compared with sTNFis, mTNFis have a significant impact on Crohn’s disease, uveitis, and psoriasis, and are associated with an increased risk of infectious complications, including opportunistic infections and tuberculosis [195,196,197,198]. TNFis were categorized into two groups: the soluble TNFi (sTNFi) group, specifically etanercept (ETN), and the monoclonal antibody TNFi (mTNFi) group. The mTNFis and sTNFis (ETN) function through different mechanisms, dependent on their unique ability to bind soluble and transmembrane TNFs and their capacity to induce apoptosis [194]. In vitro, mTNFi inhibits IFN-γ production, but sTNFi does not [199], which may suggest a difference in their susceptibility to bacterial and viral infections.

Interestingly, the clinicopathological features of RA-LPD varied between the MTX plus mTNFi and MTX plus sTNFi (ETN) groups [191]. In the MTX and sTNFi (ETN) group, CRP levels at LPD onset were higher than those in the MTX monotherapy group, and the rates of HL-like lesions, MALToma, and primary submandibular lesions increased. Additionally, the proportion of HL-like lesions was higher in the MTX group than in the mTNFi and MTX alone groups. However, the age of LPD onset increased, LDH levels decreased, the duration between the first MTX dose and the onset of LPD shortened, and the frequency of EBER-1 positive cases increased.

Patients with DLBCL treated with RTX plus CHOP therapy have an increased risk of HL compared to the general population [144]. As previously described, patients with RA who receive RTX have a high incidence of HL and MM [101]. Regarding ABT, the MTX plus ABT group showed a higher occurrence of T-LPD than the MTX-only group in our database.

TCZ is an IL-6 antagonist. IL-6 plays a role in DLBCL pathogenesis, particularly in the activated B-cell type, via the STAT3 signaling pathway [155]. Blockade of the IL-6 signaling cascade by TCZ may lower the risk of re-growth after spontaneous regression (SR), resulting in a favorable prognosis. RTX is a chimeric monoclonal antibody that targets the CD20 molecules expressed on the surface of B-cells. The MTX plus TCZ group exhibited elevated sIL-2R levels. TCZ increases the frequency of bladder and cervical cancers while decreasing the incidence of endometrial cancer [101].

#### 4.1.3. Targeted Synthetic DMARDs (tsDMARDs) and RA-LPD

Concerning JAKis, while numerous reports have suggested that there is no significant rise in the incidence of malignancies associated with JAKis [162,163,164,165,166,167,168,169], Mariette et al. noted that the age- and sex-adjusted SIR of lymphoma was 2.62 (95%CI: 1.58–4.09) [200]. This has been observed in patients treated with JAKis for the myelofibrosis associated with aggressive lymphoma [201]. Furthermore, patients with RA treated with JAKis were more often positive for ACPA and RF [200]. Harada et al. indicated that the lymphoma occurring in patients with RA undergoing treatment with JAKis was aggressive B-cell lymphoma [188].

### 4.2. EBV Status and RA-LPD

RA-LPD exhibited a higher EBER-1 positivity rate (18.4%) than sporadic LPD (51.8%). Moreover, MTX monotherapy (50.3%), MTX plus TAC (59.4%), and MTX plus TNFi (65.2%) demonstrated higher EBER-1 positivity rates than those in the naïve group. Additionally, the combined MTX plus TNFi treatment group had a higher EBER-1 positivity rate than the MTX monotherapy group [34]. Furthermore, among TNFi, mTNFis showed a higher EBER-1 positivity (65.4%) than sTNFis (ETN) (47.9%) [191]. Patients treated with mTNFis are more susceptible to EBV infection, which may result in a higher proportion of EBER-1-positive patients compared to those treated with sTNFis (ETN). In patients with RA, blood EBV antibody titers have been reported to be higher than those in the general population [202]. Moreover, it has been noted that the administration does not increase EBV antibody titers in the blood [203]. This discrepancy may arise from geographic differences in EBV positivity, highlighting the need for further studies.

### 4.3. Time Trends of RA-LPD According to Changes in Anti-Rheumatic Agents

Our large-scale study assessed the statistical differences in the characteristics of RA-LPD over time [34]. Concerning anti-RA agents administered before LPD onset, naïve cases were less frequent in the 2010s-LPD group than in the 2000s-LPD group. Conversely, MTX, TAC, TNFis, and ABT were used more frequently in the 2010s-LPD group than in the 2000s-LPD group. The estimated cumulative and weekly MTX doses in the 2010s-LPD group were higher than those in the 2000s-LPD group.

The age at RA-LPD onset was higher in the 2010s-LPD group than in the 2000s-LPD group. Immunosuppressive markers, including P-LPD and EBER-1, were increased during the later stages. The incidence of NK/T-cell lymphoma and MALToma was significantly lower in the later period [34]. In contrast, when comparing our previous and most recent publication, the prognosis for RA-LPD was worse than that for sporadic LPD in the 2007 report [179], but it reversed and improved in the 2024 report [34]. In the former case, the proportion of DLBCL was significantly higher, whereas in the latter, the significant difference disappeared.

Furthermore, the report stated that the incidence of bladder cancer, cervical cancer, prostate cancer, MM, DLBCL, multiple myeloma, and HL have increased significantly. In contrast, the incidence of pancreatic, breast, and endometrial cancers is low [101].

## 5. Discussion

This review describes the effects of various immunosuppressants and immunomodulators administered before the onset of malignancies, particularly PT-LPD and RA-LPD, as well as their changing patterns over time. Recent advances in immunosuppressants and immunomodulators have significantly improved the quality of life (QOL) of organ transplant recipients and patients with RA, leading to better long-term survival while also addressing the need to prevent malignant tumor development. As immunosuppressants and immunomodulators continue to evolve, further research is necessary to identify the optimal therapy and dosage that balances the maintenance of the transplanted organ’s function and the QOL of RA patients with a reduction in the risk of malignancy. The weaknesses of this review article include the content of the published reports, such as past studies, limited statistical analysis, small sample sizes, restricted geographic areas, insufficient verification, and low risk factors. However, essential trends that might otherwise be concealed in a large-scale meta-analysis can be identified. We believe that addressing these minor factors is important.

Overall, building evidence regarding the influence of drugs is challenging. Furthermore, determining which drug is responsible for an effect is particularly difficult when multiple drugs are administered to a single patient, considering the pharmacological synergy of all the drugs involved. The dataset from the British Society for Rheumatology Biological Registry of Rheumatoid Arthritis (BSRBR-RA) used in this study indicated that over 50% of patients who started their first TNFi later switched to alternative agents [125,204]. Prior reports examining the relationship between RA drugs, especially TNFis, and RA-LPD pathology failed to yield significant results because of inconsistencies in the group definitions and the extent of the information studied [130,189,194]. There was also potential bias in the follow-up methods, observation periods, data collection techniques, and number of cases evaluated.

In our database, 35% of patients with RA-LPD received multiple drugs, either simultaneously or at different time points. Nonetheless, significant results were obtained owing to the large number of cases, the accumulation of detailed information on the cases, and the division of patients into non-overlapping 1:1 groups, coupled with the statistical analysis of a vast quantity of data [34]. It would be intriguing to see the outcomes of earlier reports that utilized the same classification methods but did not produce significant results.

## 6. Conclusions

The trends in malignancies arising in patients treated with immunosuppressive/immunomodulatory agents are continually evolving. With advancements in medicine, understanding the various characteristics and temporal changes in immunosuppressive malignant tumors, particularly LPD, is beneficial for daily clinical practice and may lead to significant medical breakthroughs, such as the development of new drugs and improved monitoring methods for cancer treatment. Immunosuppressive malignancies including LPDs are continuously changing their characteristics due to the effect of the immunosuppressive/immunomodulatory agents.
